# Ganglioside Micelles Affect Amyloid β Aggregation by Coassembly

**DOI:** 10.1021/acschemneuro.3c00524

**Published:** 2023-12-05

**Authors:** Jing Hu, Sara Linse, Emma Sparr

**Affiliations:** † Division of Physical Chemistry, Lund University, SE-22100 Lund, Sweden; ‡ Division of Biochemistry and Structural Biology, Lund University, SE-22100 Lund, Sweden

**Keywords:** amyloid β, GM1 micelle, microfluidic diffusional sizing, microscopy, coassembly, kinetics

## Abstract

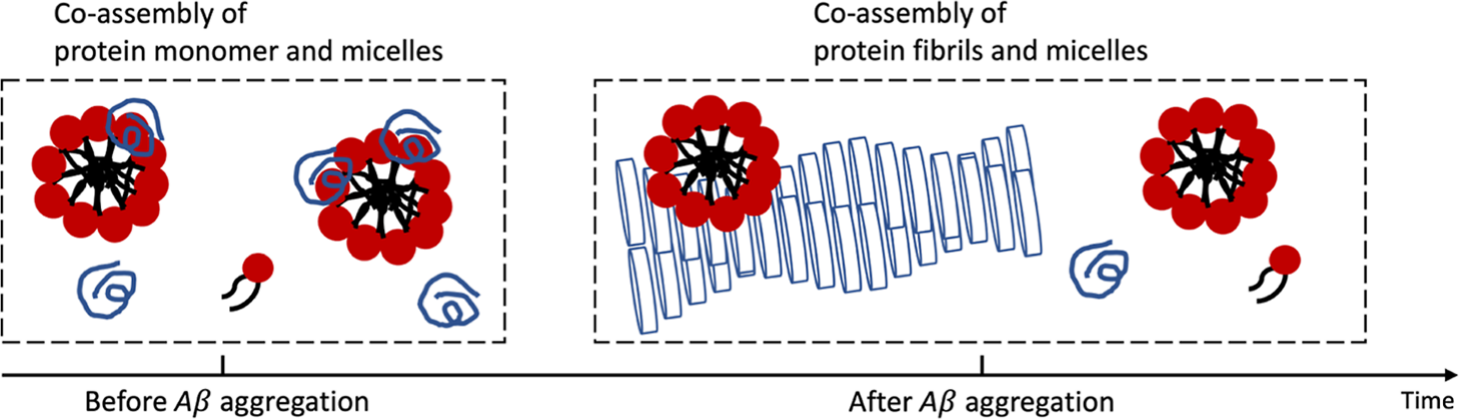

Amyloid β peptide (Aβ) is the crucial protein component of extracellular plaques in Alzheimer’s disease. The plaques also contain gangliosides lipids, which are abundant in membranes of neuronal cells and in cell-derived vesicles and exosomes. When present at concentrations above its critical micelle concentration (cmc), gangliosides can occur as mixed micelles. Here, we study the coassembly of the ganglioside GM1 and the Aβ peptides Aβ40 and 42 by means of microfluidic diffusional sizing, confocal microscopy, and cryogenic transmission electron microscopy. We also study the effects of lipid–peptide interactions on the amyloid aggregation process by fluorescence spectroscopy. Our results reveal coassembly of GM1 lipids with both Aβ monomers and Aβ fibrils. The results of the nonseeded kinetics experiments show that Aβ40 aggregation is delayed with increasing GM1 concentration, while that of Aβ42 is accelerated. In seeded aggregation reactions, the addition of GM1 leads to a retardation of the aggregation process of both peptides. Thus, while the effect on nucleation differs between the two peptides, GM1 may inhibit the elongation of both types of fibrils. These results shed light on glycolipid–peptide interactions that may play an important role in Alzheimer’s pathology.

## Introduction

Ganglioside lipids were first discovered in the brain.^1^ The concentration of gangliosides in different body regions or fluids varies considerably,^2−4^ and gangliosides are found to be 10- to 30-fold more abundant in the cerebral cortex and white matter of the brain than in other human tissues and organs.^5^ Ganglioside lipids are present in many different cell types, in particular in nerve cells, where they constitute 5–10% of the total lipid mass in the plasma membrane.^6^ Monosialotetrahexosylganglioside (GM1) is one of the major kinds of gangliosides making up 13–28% by mass of the gangliosides in different adult human brain regions.^7^ Together with three other complex gangliosides, GD1a, GD1b, and GT1b, it constitutes more than 90% of the brain ganglioside mass.^5^ It is also found that the GM1 concentration changes during aging and in common neurodegenerative conditions such as Alzheimer’s disease (AD), Parkinson’s disease, and Huntington’s disease.^5^

When GM1 is added to an aqueous solution, it forms micelles rather than bilayers, which can be explained by the large and negatively charged headgroup composed of an oligosaccharide with one sialic acid moiety. In mixtures of GM1 and zwitterionic 1,2-dioleoyl-*sn*-glycero-3-phosphocholine, up to around 10 mol % GM1 can be dissolved in the phosphocholine (PC) bilayer, while at higher GM1 concentrations the PC-rich bilayer coexists with GM1-rich micelles.^8^ The solubility of GM1 in a lipid bilayer varies with the lipid composition, the bilayer phase behavior, and the total lipid concentration,^9,10^ although the overall behavior with coexisting self-assembled structures is expected for any mixture of GM1, which has a conical shape, and phospholipids of cylindrical shape.^11^ It is, therefore, a relevant scenario that ganglioside-rich micelles can be present in the brain tissue at locations with high ganglioside contents.

Micelles are fundamentally different from bilayer membranes in several ways: Micelles are small dispersed particles freely diffusing in an aqueous solution, while membranes are normally larger entities. Micelles are dynamic structures due to their relatively high GM1 solubility in water. Micelles are also efficient at solubilizing hydrophobic and amphiphilic molecules. These properties can all impact the assembly and transport processes of different biomolecules,^11^ including processes associated with protein aggregation and amyloid formation.^12−15^

**Figure 1:**
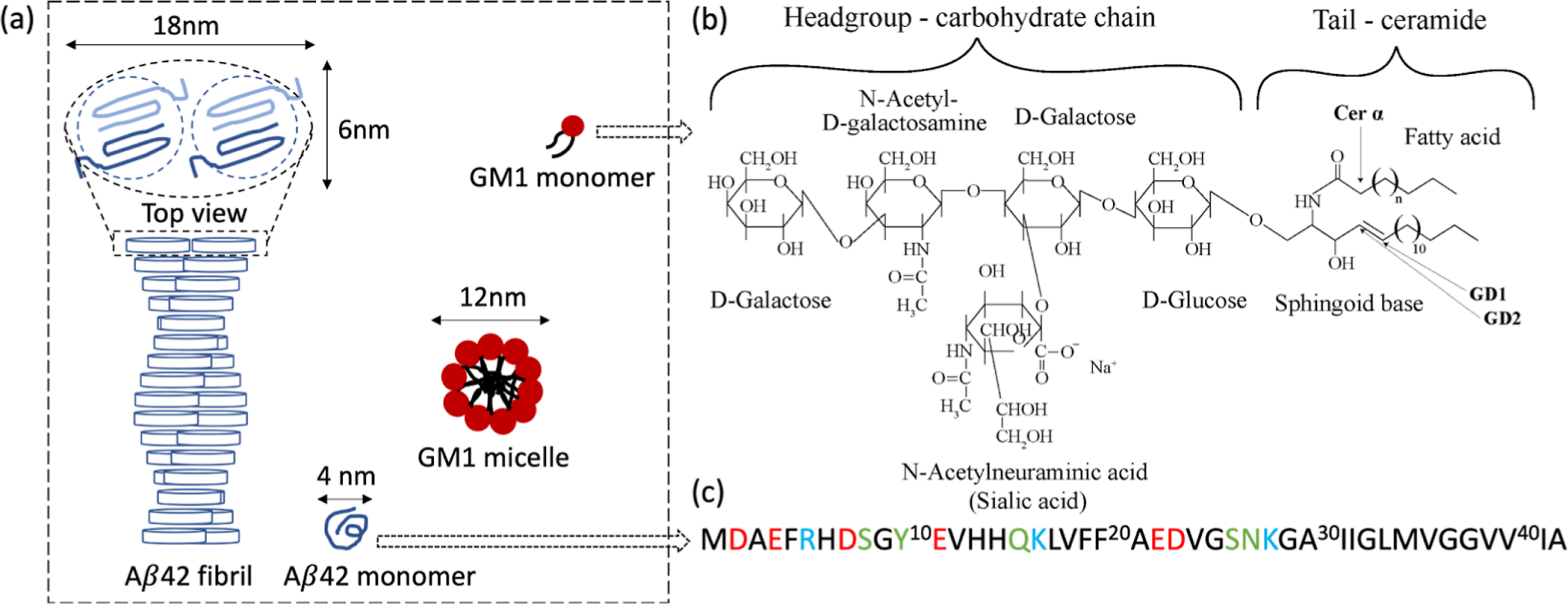
Schematic illustration of Aβ monomer, Aβ fibril, GM1 monomer, and GM1 micelle. (a) Cartoons with blue lines represent Aβ monomers and blue cylinders represent the plane of Aβ monomers in the fibril. (b) Molecular structure of ganglioside GM1.^8^ (c) Amino acid sequence of Aβ42 used in this study. Red, blue, green, and black denote negatively charged residues, positively charged residues, polar neutral residues, and nonpolar residues, respectively.

Amyloid beta peptide (Aβ) is produced through the proteolytic processing of the transmembrane protein amyloid-β precursor protein (APP). Extracellular deposition of Aβ amyloid aggregates is a hallmark of AD. The main cleavage products of APP are the 40-amino-acid residue peptide Aβ40 and the 42-amino-acid residue peptide Aβ42^16^ (Figure 1a), although a large set of length variants are found in vivo.^17−19^ There is much evidence that brain Aβ aggregation is an early and central pathophysiological alteration that drives other AD-related processes.^20^ Therefore, it is crucial to understand the molecular mechanism of Aβ self-assembly and to elucidate which factors govern or counteract the process.

Many in vivo studies have indicated the biological significance of GM1 in AD. For example, GM1-bound Aβ was found in human cerebrospinal fluid.^21^ In addition, imaging mass spectrometry studies have shown that GM1 gangliosides are enriched and colocalized with Aβ40 in the core region of mature plaques,^22,23^ indicating attractive interactions between GM1 and Aβ. It is possible that the uptake of GM1 into plaques is facilitated by the relatively high water solubility of GM1 as compared to most other membrane lipids and the relatively high diffusional transport of the GM1 micelles. In analogy with detergent micelles, GM1 micelles may act to solubilize hydrophobic and surface-active molecules, e.g., Aβ, increasing their apparent solubility. Previous in vitro studies have indicated that GM1-containing membranes interact with Aβ and influence Aβ aggregation kinetics.^24−31^ Several of these studies used GM1-containing membranes with a rather high fraction of gangliosides in the lipid mixtures. Still, the possibility of coexisting GM1-rich micelles and their influence on Aβ aggregation were not specifically addressed.

In the present study, we have investigated the interaction and association between Aβ and GM1 micelles, focusing on three subquestions: (i) do Aβ monomers coassemble with GM1 micelles? (ii) Is there any coassembly between GM1 and Aβ in amyloid fibrils? (iii) How does the presence of GM1 micelles influence the amyloid formation process and rates of the underlying steps? To answer these questions, we investigated a system composed of Aβ (Aβ40 or Aβ42) and GM1 micelles at different stages of the amyloid formation process, including the initial and final stages. We have used a range of complementary techniques, including microfluidic diffusional sizing (MDS), confocal fluorescence microscopy, cryogenic transmission electron microscopy (cryo-TEM) as well as fluorescence spectroscopy.

## Results

Figure 1 shows the system of this study and indicates the typical length scales of the components. The hydrodynamic radius of the random coil Aβ42 monomer is ∼2 nm.^32^ Aβ fibrils are observed to be polymorphic when extracted from complex environments such as brain tissue.^33^ Aβ42 fibrils prepared in aqueous phosphate buffer at pH 7.4–8.0 are found to be monomorphic^34^ with the structural agreement between investigators;^35^ Aβ42 fibrils prepared in aqueous phosphate buffer at pH 7.4 at 37 °C have an elliptical cross-section with a major axis of ∼18 nm and a minor axis of ∼6 nm.^36^ The Aβ40 fibril seems to have a bigger cross-section compared to that of Aβ42, with at least 4 filaments.^37^ Previous studies have shown that the GM1 micelles have a spherical shape with a radius of ∼6 nm over a wide range of GM1 concentrations (1–15 mM).^8,38,39^ Reported values of the critical micelle concentration (CMC) of GM1 vary considerably within the 10^–9^ to 10^–6^ mM range.^40−43^ For the present GM1 batch, we showed that CMC is below 5 μM (Figure S5) from pyrene fluorescence spectroscopy measurements. The CMC of GM1 is thus clearly below the lowest GM1 concentration used in the current experiments.

### Average Protein Size in the Presence of GM1 Micelles

To investigate the coassembly between Aβ monomers and GM1 (the initial stage of the aggregation process), we used MDS to measure the diffusion rate of Aβ or GM1 micelles. MDS monitors the diffusion of fluorescent species, which can be used to estimate the average hydrodynamic radius (*r*_H_) of the labeled molecules.^44^ A cysteine mutant (S8C) of Aβ42 was covalently labeled with Alexa-647 (Alexa-Aβ42) using maleimide chemistry. Position 8 was chosen for labeling because it has been shown to give similar aggregation kinetics and fibril morphology as wild-type Aβ42.^45^ The labeled protein (20 nM) was mixed with varying amounts of unlabeled GM1 (12–1000 μM) above the CMC of GM1 (Figure S5). The Aβ42 concentration was chosen to not exceed the reported Aβ42 solubility (∼20 nM),^46^ meaning that Aβ42 self-aggregation at this concentration can be neglected, at least for the time frame of the experiment (5 min). The average diffusion rate of Alexa-Aβ42 was measured for each sample using MDS with excitation at 650 nm. In separate experiments, the diffusion of GM1 was studied using GM1 doped with 0.5 mol % NBD-PE, which is a fluorescently labeled lipid containing 18 carbons in each of the two acyl chains. NBD-PE has low solubility in water and is thus solubilized into GM1 micelles. The average diffusion rate of GM1 micelles was measured by MDS with excitation at 488 nm.

**Figure 2:**
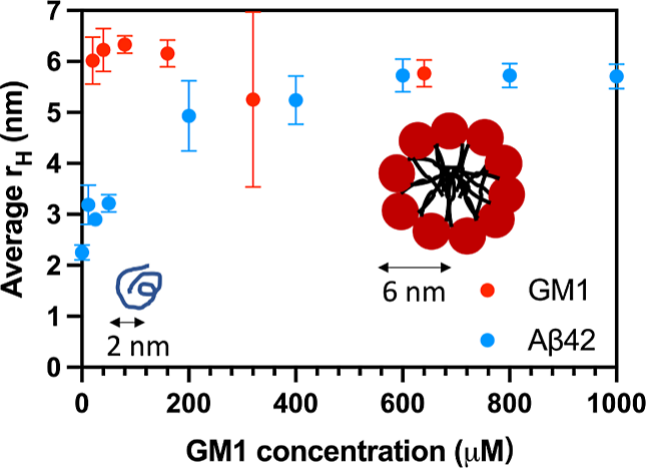
MDS data for diffusion in mixtures of Aβ42 and GM1. Average hydrodynamic radius of 20 nM Alexa647-Aβ42 in the absence and presence of 12–1000 μM unlabeled GM1 (blue) and of 12–1000 μM GM1 containing 0.5 mol % NBD-PE (red) is plotted as a function of GM1 concentration. The cartoons are schematic illustrations of the Aβ monomer and GM1 micelle.

The data in Figure 2 show that the average *r*_H_ of the GM1 micelles is ∼6 nm, which is in agreement with reported values.^8^ The diffusion rate of the micelles is almost constant over the range of GM1 concentrations studied. This implies that the GM1 aggregation number remains more or less the same over this concentration range. The apparent size of Alexa-Aβ42 is around 2 nm in the absence of GM1, which is consistent with previous measurements.^32^ The addition of GM1 to the solution containing Alexa-Aβ42 leads to an increase in the average *r*_H_ until it reaches a stable value of around 6 nm at 600 μM or more GM1. In other words, the apparent size of Alexa-Ab42 in the presence of GM1 micelles is close to that of the GM1 micelles. Taken together, the data in Figure 2 imply the coassembly of Alexa-Aβ42 monomers with GM1 in mixed micelles.

### Coassembly of Lipids and Fibrils Measured by Microscopy

After concluding that GM1 coassembles with Aβ monomers, we now turn to the other end of the aggregation process, that is, the final state amyloid aggregates. We have investigated whether there is a coassembly of GM1 and Aβ in amyloid fibrils employing a combination of microscopy techniques. First, we used confocal microscopy to study the colocalization of proteins and lipids. In these experiments, the protein was unlabeled, and the amyloid-specific oligothiophene pFTAA^47^ was added to detect protein amyloid aggregates. The lipid analog Atto-DPPE was added to the GM1 micelles. Atto-DPPE has a very low solubility in water and is solubilized in the micelles. In this experiment, we used a protein concentration (5 μM), for which amyloid aggregation occurs within 2 h (Figure S6), and a GM1 concentration (800 μM) that is more than 100 times the CMC, meaning a high concentration of micelles in the sample. Samples were incubated for at least 3 days before imaging.

**Figure 3:**
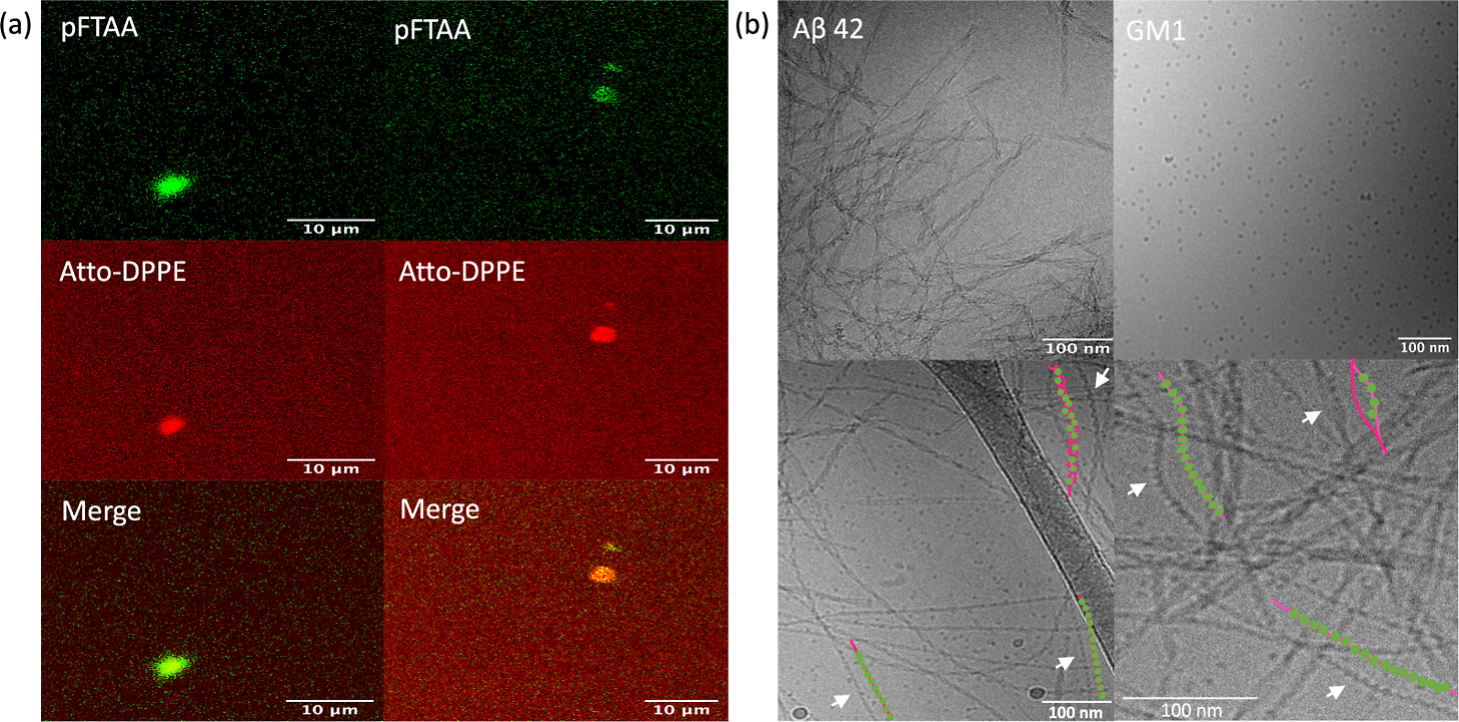
Confocal microscopy images of solutions containing 800 μM GM1 and 5 μM Aβ42, incubated for 3 days. pFTAA (0.75 μM) was used to detect aggregated protein in the green channel, and Atto-DPPE (4 μM) was used to detect GM1 micelles in the red channel. Merged images of green and red channels show colocalization (a). cryo-TEM images of fibrils formed in a solution with 5 μM Aβ42 after 5 days of incubation, 800 μM GM1, and the mixture of 800 μM GM1 (containing 8 μM Atto-DPPE) and 5 μM Aβ42 after 5 days of incubation (b). The white arrows indicate cases in which lipids decorate Aβ fibrils. The schematic illustrations of structures indicated by the white arrow are shown by purple lines and green dots representing amyloid fibrils and micelles, respectively.

Confocal fluorescence microscopy images from the samples containing both GM1 and Aβ42 show pFTAA overlapping with Atto-DPPE, implying colocalization of protein and lipids on a μm to sub-μm length scale (Figure 3a). In the confocal fluorescence images, we cannot resolve individual fibrils and only observe lumps of fibrils. In control experiments, the lipid analog Atto-DPPE was exchanged for NBD-PE, which has longer carbon chains and therefore even lower solubility in water. The confocal microscopy images confirm the colocalization of lipids with protein clusters (Figure S2). However, the detailed structure of such coassemblies cannot be revealed by optical microscopy, thus motivating studies at even higher resolution.

To gain a more detailed view of the structure of the coassemblies, we used cryo-TEM to image samples at the nanometer level. The sample preparation and composition were similar to those for optical microscopy, except no fluorophores were used.

As shown in Figure 3b, pure Aβ42 fibrils appear to have a rather smooth surface along the sides of the fibrils. Samples composed of 800 μM GM1 alone appear to be monodisperse and micelles are observed as small dots with a radius of ∼5 nm, which is directly measured from the cryo-TEM images (Figure S5), and consistent with the results from the MDS experiments (Figure 2) and previous reports.^8,48^ In the samples with fibrils formed in the presence of GM1, we observe Aβ42 fibrils decorated by small dots but also some small dots free in the surrounding solution. These small dots have a similar size, shape, and contrast to those of the pure GM1 micelles in Figure 3b. The decorated fibrils are highlighted in the images by white arrows. These results indicate that GM1 micelles adsorb on the Aβ fibril surface.

### Aβ Aggregation Kinetics Influenced by GM1

The experimental data above infer the coassembly of Aβ and GM1 at both end states of the aggregation process, i.e., both with Aβ42 monomers and fibrils. Next, we investigated how GM1 may interfere with the process of Aβ42 amyloid formation.

To follow the aggregation process, we added pFTAA to the samples containing 3 μM protein and GM1 at different concentrations and monitored the fluorescence of pFTAA at 520 nm. pFTAA was chosen as the protein-aggregation-specific dye because its fluorescence signal is not sensitive to the presence of GM1, whereas fluorescence enhancement by GM1 precludes the use of thioflavin T (Figure S4). The pFTAA concentration was optimized to 1.5 μM, at which concentration the intensity difference before and after protein aggregation reaches a maximum (Figure S6) and shows a linear response versus fibril concentration.

**Figure 4:**
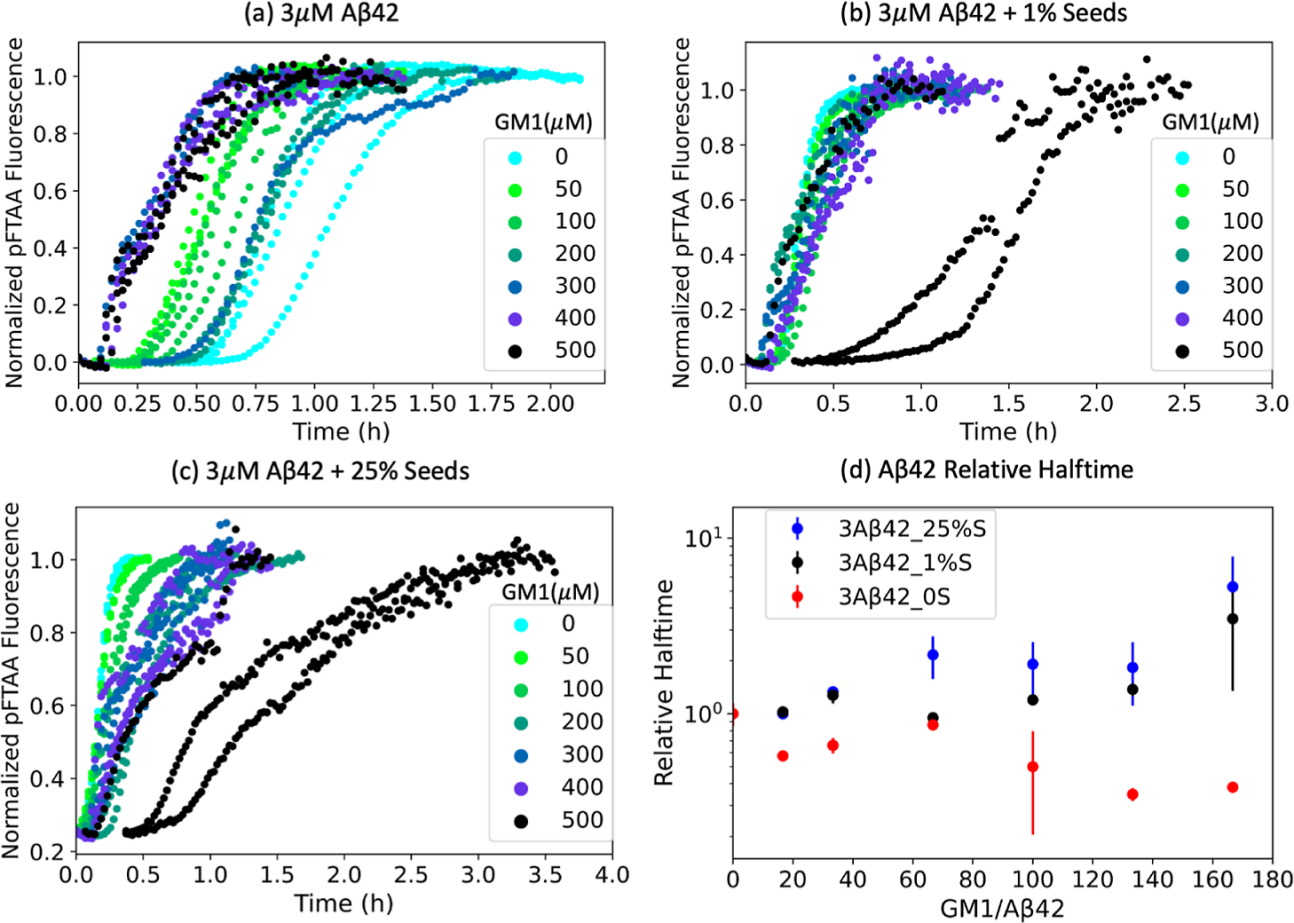
Aggregation kinetics of 3 μM Aβ42 without seeds (a), with 1% seeds (b), or with 25% seeds (c) in the presence of different concentrations of GM1. Relative half-time extracted from these kinetic curves plotted against GM1/Aβ42 ratio (d). All these kinetic curves were monitored by pFTAA (1.5 μM) fluorescence intensities. Relative halftime is the halftime normalized by the halftime at a lipid/protein ratio of 0. Halftime is taken at the time when the normalized fluorescence is 0.5 for 0 or 1% seeds and 0.6 for 25% seeds.

As shown in Figure 4a, the aggregation of nonseeded Aβ42 was accelerated by the addition of GM1 at all concentrations investigated. To get insights into the mechanism behind the accelerating effect of GM1, we also studied the aggregation kinetics at different seed and GM1 concentrations (Figure 4b–d). Experiments were performed at light seeding (1%) to bypass the primary nucleation, and at heavy seeding (25%) to bypass both primary and to a large extent secondary nucleation.^49^ The experiments performed at low seed concentrations primarily provide information about the effects of GM1 on secondary nucleation and elongation, while the experiments performed at high seed concentrations mainly report on how GM1 affects the elongation process. As shown in Figure 4b–d, the seeded Aβ42 aggregation is retarded when GM1 is present at high concentrations both in light and heavy seeding experiments. On the other hand, no strong effects are observed at the lower GM1 concentrations.

### Co-Assembly of Aβ40 and GM1

The experiments above revealed the coassembly of Aβ42 and GM1. Next, we investigated whether the same behavior is also present for the other main Aβ variant Aβ40, using the same experimental approach as that in the GM1 and Aβ42 studies.

**Figure 5:**
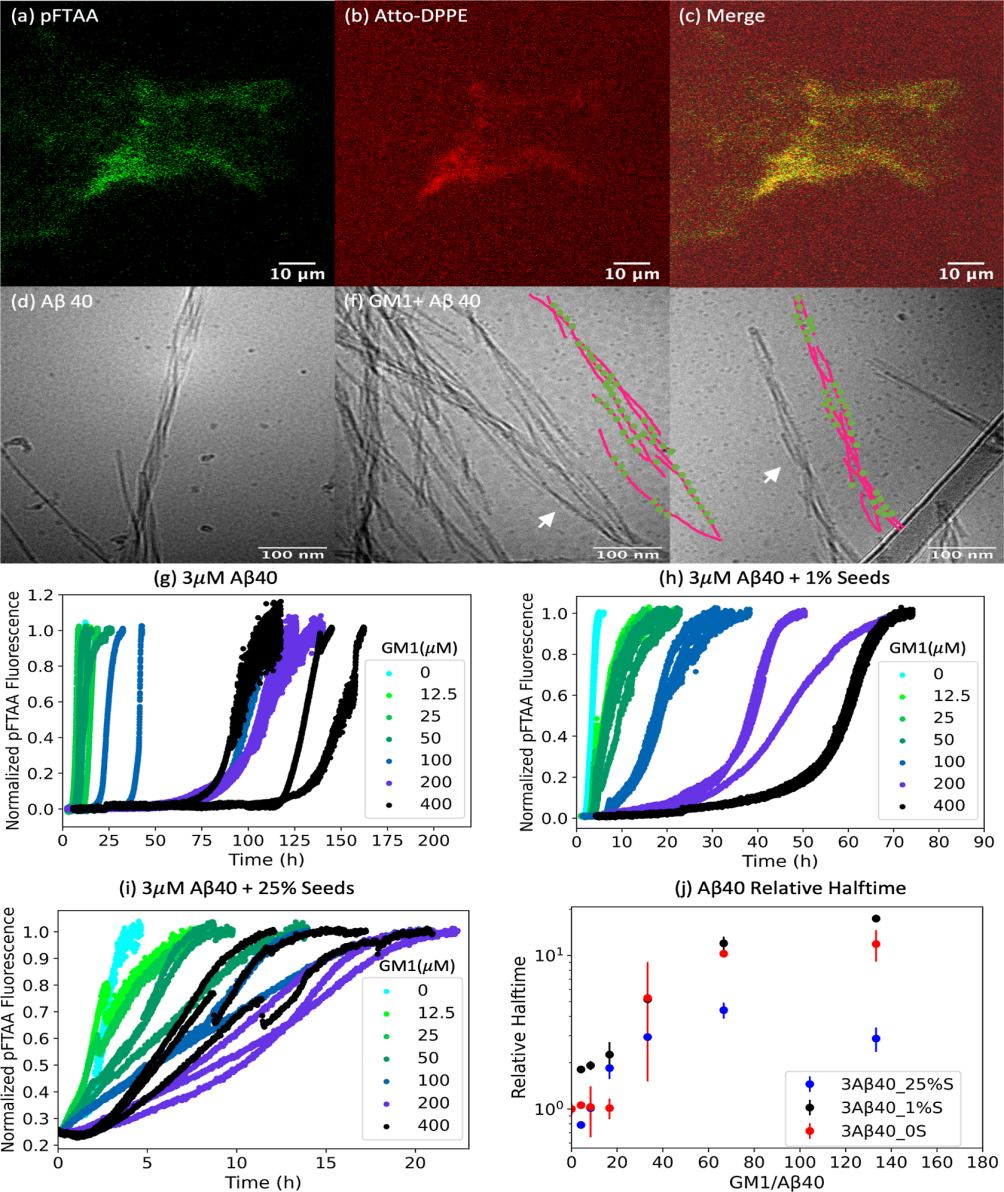
Confocal microscopy images of solutions containing 800 μM GM1 and 5 μM Aβ40, incubated for 7 days, pFTAA (0.75 μM, green) is used to detect aggregated protein (a), atto-DPPE (4 μM, red) is used to detect GM1 micelles (b), merged images from green and red channel show colocalization of pFTAA and atto-DPPE (c). cryo-TEM images of fibrils formed in a solution with 3 μM Aβ40, incubated for 7 days (d), and the mixture of 500 μM GM1 and 3 μM Aβ40, incubated for 7 days (e,f). The schematic illustrations of white arrows pointed structures are shown by the purple lines and green dots representing amyloid fibrils and micelles, respectively. Aggregation kinetics of 3 μM Aβ40 without seeds (g), with 1% seeds (h), or with 25% seeds (i) in the presence of different concentrations of GM1. The dips in fluorescence intensity visible in traces are likely due to instrument artifacts such as mechanical disturbance of the plate. Relative halftime extracted from these kinetic curves plotted against GM1/Aβ40 ratio (j). All these kinetic curves were monitored by pFTAA (1.5 μM) fluorescence intensities.

The confocal microscopy images show colocalization of lipids and Aβ40 amyloid aggregate clusters (Figure 5a–c). It has previously been observed that Aβ40 fibrils display a longer pitch (node-to-node distance) than the more highly twisted Aβ42 fibrils,^50^ which is in line with the fibril morphologies shown in Figures 3b and 5d. In the samples with fibrils formed in the presence of GM1 (Figure 5e,f), we again observe fibrils decorated with small dots that are likely fibril-associated micelles.

From the aggregation kinetics experiments (Figure 5g), we conclude that GM1 retards Aβ40 aggregation above lipid–protein ratios of 40, which is opposite to the findings obtained for Aβ42 at the same protein and lipid concentrations. Cryo-TEM images for samples withdrawn at intermediate time points (15 h, Figure S6) again show decorated Aβ40 fibrils. Finally, we studied the effects of GM1 on seeded samples (Figure 5h–j). GM1 is found to delay aggregation for both lightly and heavily seeded Aβ40 reactions.

## Discussion

In this work, we have characterized the interaction between Aβ and GM1 micelles before and after aggregation. We observe the coassembly of protein monomers and micelles before aggregation as well as the coassembly of protein fibrils and micelles at the end of the aggregation reaction. We also find that the presence of GM1 interferes with the Aβ aggregation process, which is likely related to the observed coassembly processes. For example, the coating of the fibril surface will affect the reactions on the fibril surface, thus influencing the aggregation process. The interaction of Aβ monomers and GM1 micelles can also influence the initiation of the aggregation process.

From the MDS results, we conclude that Aβ and GM1 are present together in objects of similar size to the pure GM1 micelles, indicating the formation of mixed lipid–protein micelles. However, MDS measures the average diffusion providing no information at a single micelle or single protein level. From these experiments, we can thus not determine the proportion of lipid and protein in the micelles, or the location of the protein in the mixed micelles. There are several studies in the literature on the structures of mixed micelles formed by Aβ and other lipids or detergents. Studies using nuclear magnetic resonance spectroscopy have suggested that Aβ40 orients in parallel pairs on the hydrophobic/hydrophilic interface of lyso-GM1 micelles, with two α-helices comprising residues 14–24 and 31–36, as well as the C-terminal segment, in contact with the hydrophobic micelle interior with the remaining regions exposed to the hydrophilic environment.^51^ These findings are in line with findings for Aβ and other micellar systems, including sodium dodecyl sulfate (SDS), where hydrophobic regions reside in SDS micelles, while the hydrophilic N-termini are to the aqueous environment.^52,53^

There are several mechanisms by which the formation of mixed lipid–protein micelles may influence the amyloid aggregation process. The depletion of protein monomers from the solution may retard all nucleation and growth processes, as monomers are reactants in all of the aggregation reaction steps. The accumulation of proteins in the micelles, in the hydrophobic core or at the micelle interface, may facilitate nucleation for protein aggregation, depending on the local protein concentration and organization of proteins in the micelles. The formation of Aβ-GM1 mixed micelles (Figure 2) may thus cause both acceleration and retardation of the aggregation process.

The fact that we observe lipids also in the final state amyloid fibrils implies not only that the GM1 micelles are providing a catalyzing surface but also that the lipids, in some way, are involved in the aggregation process. The cryo-TEM images suggest associated micelles along the side of the fibrils (Figures 3b and 5e,f). Similarly, coated Aβ fibrils and altered fibril morphology were observed in the presence of lipid vesicles containing GM1.^54^ The decoration of amyloid fibrils with GM1-containing assemblies may influence all processes that occur at the fibril surface including secondary nucleation and elongation. For the present systems, micelles may block the sides and the ends of the fibrils. It is also possible that the interaction between Aβ oligomers and micelles influences the protein aggregation process, for example, the steps of conversion and detachment of Aβ oligomers from the fibrils.

The results from Aβ40 and Aβ42 aggregation reveal that GM1 retards Aβ40 aggregation both in the absence and presence of seeds, while Aβ42 aggregation is catalyzed in nonseeded reactions and retarded in seeded ones. One possible explanation for the discrepancy between the two peptides may be that GM1 accelerates the primary nucleation of Aβ42 by forming mixed micelles. In contrast, for less hydrophobic Aβ40, the drive for forming mixed micelles is likely lower. On the other hand, in the seeded samples where primary nucleation is bypassed, the addition of GM1 leads to slower aggregation as compared to the protein-only samples for both Aβ40 and Aβ42. A possible explanation for this is that GM1 interferes with the sites for secondary nucleation or elongation, thus slowing down processes that rely on these sites.

From a broader perspective, this study hints at the important roles that micelles can play in complex biological environments, including ganglioside-rich neural cells in the brain. For example, micelles may stabilize diffusible forms of Aβ including monomers or oligomers by solubilization. Micelles may also promote the transport of these small mono- and oligomeric Aβ species. These factors might contribute to the uptake of GM1 into the core of Aβ plaques.^22^ A recent study using cryogenic fluorescence microscopy and in-tissue cryo-electron tomography also found that smaller lipid vesicles or exosomes are present in the core of plaque, while bigger constituents are found at the edge. The in vivo findings together with the basic characterization of coassembly of amyloid-forming proteins and micelle-forming lipids,^12−15,51,52^ including the present study, may thus spread light on the potentially important role of micelles in biological environments.

## Materials and Methods

### Materials

GM1 ganglioside (from the ovine brain, sodium salt) was purchased from Avanti Polar Lipids (Alabaster AL). Lyophilized Atto-647 1,2-dipalmitoyl-*sn*-glycero-3-phosphoethanolamine (DPPE-647) was purchased from ATTO-TEC GmbH. 18:1 NBD-PE ammonium salt (1,2-dioleoyl-*sn*-glycero-3-phosphoethanolamine-*N*-(7-nitro-2–1,3-benzoxadiazol-4-yl)) was purchased from Avanti Polar Lipids (Alabaster AL). pFTAA was a kind gift by K. Peter R. Nilsson, Linköping University.^47^ Phosphate buffer of 20 mM sodium phosphate, 0.2 mM ethylenediaminetetraacetic acid (EDTA), and 0.02% NaN_3_, at pH 7.4, was used to dissolve all proteins and lipids in all experiments (except protein expression and purification steps). The buffer chemicals were of analytical grade and Milli-Q water was used to prepare all buffer solutions.

### GM1 Sample Preparation

Stock solutions of GM1 were prepared in chloroform/methanol 2:1 (v/v) and stored at −20 °C. For GM1 samples containing fluorophores, the fluorophores were stored in chloroform/methanol 2:1 (v/v) and then mixed with GM1 samples in the same solvent. Before use, the solvent was evaporated under a stream of dry N_2_ gas, and a thin lipid film was obtained. To ensure complete solvent evaporation, the lipid film was dried overnight in a vacuum chamber. The lipid films were rehydrated with a phosphate buffer. The same approach was used for GM1 alone and for GM1 containing trace amounts of fluorophores. The incubation temperature for all of the experiments was 37 °C.

### Recombinant Aβ40 and Aβ42 Expression and Purification

Aβ(M1-42) peptide, here called Aβ42, MDAEFRHDSGYEVHHQKLVFFAEDVGSNKGAIIGLMVGGVVIA, was expressed in *Escherichia coli* BL21(DE3)pLysS Star and purified using a combination of sonication, centrifugation, anion-exchange chromatography, and gel filtration chromatography, as described before.^55,56^ Such purified Aβ42 monomers were aliquoted, lyophilized, and stored at −20 °C until further usage.

Aβ(1–40) peptide, here called Aβ40, DAEFRHDSGYEVHHQKLVFFAEDVGSNKGAIIGLMVGGVV, was expressed in *E. coli* in fusion with the Npro autoprotease mutant called EDDIE^57^ and purified according to the following procedure. EDDIE-Aβ40 was expressed from a Pet3a plasmid (purchased from Genscript, Piscataway, New Jersey) in *E. coli* BL21(DE3)pLysS Star.

The purification of Aβ40 can be divided into 5 steps as previously reported for Aβ42:^49^ anion-exchange (DEAE column), autocleavage, dialysis, anion-exchange (batch purification), and size exclusion chromatography. (1) Anion-exchange (DEAE column): cell pellet from 4L culture was sonicated with 30 pulses (1s on/off, dynamic amplitude to 10%) five times in 80 mL of sonication buffer (10 mM Tris/HCl, 1 mM EDTA, pH 8.5, DNase for first two sonications), with centrifugation at 18,000*g* for 7 min and removal of supernatant between sonications. The resulting solution was loaded onto 2 × 20 mL DEAE-sepharose FF columns in tandem (pre-equilibrated in 4 M urea, 10 mM Tris, 1 mM EDTA, 1 mM DTT, pH 8.5) and the protein was eluted with a 0–0.4 M NaCl gradient in the same buffer. The eluted fractions were examined using UV absorbance and SDS–PAGE. (2) Autocleavage: fractions containing EDDIE-Aβ40 were diluted 15 times with 1 M Tris, 1 mM EDTA, 5 mM DTT, at pH 7.9, total volume 1.5 L, and left at 4 °C for 48 h. (3) Dialysis: the solution was dialyzed in 3.5 kDa MW cutoff dialysis bags three times against 10 L of buffer of 5 mM Tris/HCl, 0.5 mM EDTA, at pH 8.5. (4) Anion-exchange (batch purification): the solution was incubated with 50 g *Q*-sepharose big beads (GE Healthcare, equilibrated in 10 mM Tris/HCl, 1 mM EDTA, pH 8.5) for 0.5 h at 4 °C under stirring, and collected on a funnel, washed with 200 mL of equilibration buffer, and eluted with 8 × 100 mL of 10 mM Tris/HCl, 1 mM EDTA, 75 mM NaCl, pH 8.5. The eluted fractions were examined using SDS–PAGE, and fractions dominated by Aβ40 monomers were lyophilized. (4) Size exclusion chromatography: lyophilized samples were dissolved in 10 mL 6 M GuHCl, 20 mM sodium phosphate, at pH 8.5, and loaded onto a Superdex 75 26/600 column (GE Healthcare, pre-equilibrated in 20 mM sodium phosphate, pH 8.5). The eluted fractions were examined using UV absorbance and SDS–PAGE. Fractions corresponding to the center of the Aβ40 monomer peak were pooled and lyophilized. Such size exclusion process was conducted twice, and the resulting monomers were further aliquoted, lyophilized, and stored at −20 °C.

Just prior to each experiment, the Aβ42 or Aβ40 monomers mentioned above were dissolved in 1 mL 6 M GuHCl, 20 mM sodium phosphate, at pH 8.5 and further isolated by SEC in filtered and degassed phosphate buffer (20 mM sodium phosphate, 0.2 mM EDTA, 0.02% NaN_3_, at pH 7.4), using a Superdex 75 Increase 10/300 GL (GE Healthcare) column. The protein concentration was determined by measuring UV absorbance at 280 nm and using the extinction coefficient of 1400 L mol^–1^ cm^–1^.

Alexa-Aβ42 was prepared as previously reported.^45^

### Microfluidic Diffusional Sizing

The Alexa647-labeled Aβ42 monomers were mixed with/without GM1 (1:1 volume) to make mixtures of 20 nM protein and 0–1000 μM GM1. Such mixtures were incubated at 37 °C for 5 min, and then 5 μL of each sample was loaded to the microfluidic channel slides and measured by Fluidity One W serum instrument (Fluidics Inc. Cambridge, UK). The instrument was set at an intermediate flow rate which allows for the diffusion of objects with a hydrodynamic radius ranging from 1.5 to 8 nm.

### Confocal Laser Scanning Microscope

The Aβ42 or Aβ40 monomers and GM1 were mixed (1:1 volume ratio) with pFTAA to make mixtures of 5 μM protein, 0.75 μM pFTAA, 800 μM GM1, and 4 μM Atto-DPPE or 8 μM NBD-PE. The mixtures were incubated at 37 °C for several days (3 days for Aβ42 mixture and 7 days for Aβ40) and then 4.5 μL of each sample was placed between a glass slide and a cover glass with a 0.12 mm silicone spacer. The sample was imaged by a confocal laser scanning microscope (CLSM, Leica SP5) operated in the inverted mode (D6000I) with a 100 ×/1.40 oil immersion objective. The temperature of the samples was controlled at 37 °C by mounting the CLSM to a thermostated enclosure. The red fluorescence of Atto-DPPE and green fluorescence of pFTAA were excited using a Helium/Neon laser at 633 nm or an argon laser at 458 nm, respectively. The contrast of all images was autoadjusted by ImageJ.

### Cryogenic Electron Microscopy (Cryo-TEM)

The Aβ42 or Aβ40 monomers with and without GM1 were incubated at 37 °C in PEGylated polystyrene plates (Corning 3881) in a plate reader for several days before they were collected for cryo-TEM experiments. The preparation of samples for cryo-TEM was performed as previously reported.^45^ The specimens were collected and plunged using an automatic plunge freezer system (Leica EM GP), which is set at a controlled chamber temperature and relative humidity. Specimens were prepared as thin liquid films on glow-discharged lacey Formvar carbon-coated copper grids (Ted Pella) and plunged into liquid ethane at −184 °C. In this way, the specimens were vitrified and adopted a glass-like state, avoiding the formation of ice crystals and thereby preserving the original microstructures. The specimens were stored under liquid nitrogen until transferred into the electron microscope (JEM 2200FS) using a Fischione model 2550 cryo transfer tomography holder. The acceleration voltage was 200 kV and zero-loss images (using an in-column energy filter) were recorded digitally with a TVIPS F416 camera using SerialEM under low dose conditions with a 10 eV energy selecting slit in place. The contrast of all images was autoadjusted by ImageJ.

### Aggregation Kinetics by pFTAA Fluorescence

The freshly purified proteins were kept on ice and mixed with GM1 and pFTAA, which were aliquoted in 96-well PEGylated polystyrene plates (half area, Corning 3881), 80 μL per well, and sealed with a plastic film to avoid evaporation. The aggregation experiments were initiated by placing the 96-well plate at 37 °C in a plate reader (FluoStar Omega). The pFTAA fluorescence was measured through the bottom of the plate every 85 s (continuous measurement through wells) using an excitation filter at 448 nm and an emission filter at 520 nm. For seeded aggregation experiments, the seeds were collected shortly after the kinetic curve had reached the plateau.

### Fluorescence Spectra of Pyrene

A stock solution of 2 mM pyrene in methanol was prepared by dissolving 1 mg of pyrene in 2.5 mL methanol. The stock was diluted 10 times by mixing 0.2 mL of 2 mM pyrene with 1.8 mL methanol. Then, a stock solution of 600 nM pyrene in buffer was made by drying 10 μL 0.2 mM pyrene overnight in a vacuum chamber, rehydrated with 3323 μL phosphate buffer, and sonicated in the water bath for 1 h for a full dissolution. Such a pyrene solution was mixed with GM1 to make a solution containing 200 nM pyrene and 200 μM GM1. The fluorescence spectra of the solution were detected by spectrometer from Cary Eclipse at 37 °C in a 1 mL cuvette. Such mixture solution was diluted by the same pyrene solution and detected using the same instrument. Pyrene was excited at 334 nm and fluorescence emission was measured from 350 to 450 nm. All spectra were subtracted by the buffer spectrum.

## Supporting Information Available

Fluorescence spectra, confocal fluorescence microscopy, cryogenic electron microscopy, and aggregation kinetics by pFTAA fluorescence (PDF)
