# Supplementary material for: Ganglioside Micelles Affect Amyloid β Aggregation by Coassembly
Source: ACS Chem Neurosci. 2023 Dec 5;14(24):4335–43. doi: 10.1021/acschemneuro.3c00524 (PMC10739608; doi:10.1021/acschemneuro.3c00524)
Supplement: Supplementary file 1 — cn3c00524_si_001.pdf [file cn3c00524_si_001.pdf]

Supporting Information: Ganglioside Micelles Affect Amyloid β Aggregation by Co-assembly

Jing Hu,[*,†] Sara Linse,[‡] and Emma Sparr[†]

†*Division of Physical Chemistry, Lund University, SE-22100 Lund, Sweden*

‡*Division of Biochemistry and Structural Biology, Lund University, SE-22100 Lund, Sweden*

E-mail: jing.hu@fkem1.lu.se

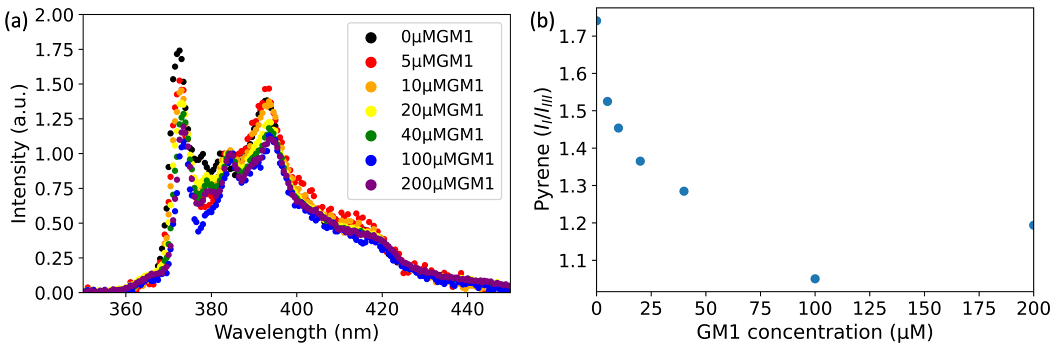

Figure S1: Fluorescence spectra of 200nM pyrene with different concentration of GM1 lipids (a). The ratio of the first (around 373 nm) and third (around 384 nm) peak from the previous fluorescence figure plotted against GM1 concentration (b). The critical micelle concentration of GM1 was detected by this pyrene assay to be lower than 5 μM, because pyrene spectrum was already altered with the addition of 5 μM GM1 micelles, indicating the existence of micelles.

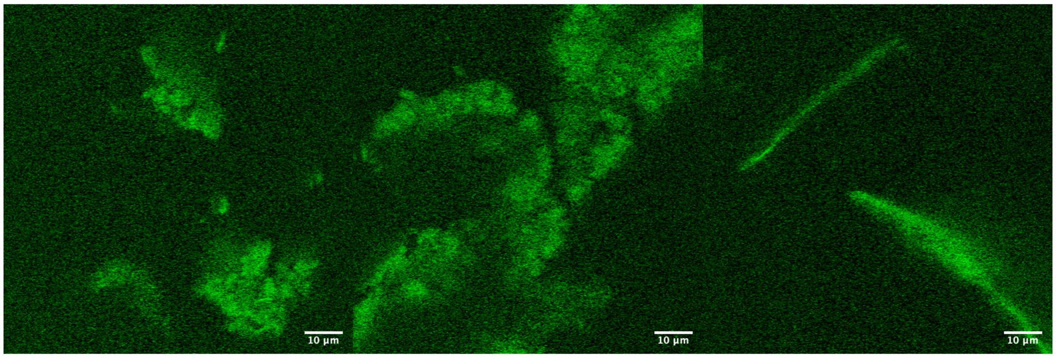

Figure S2: Confocal microscopy images of solutions containing 800 μM GM1 and 5 μM Aβ42, incubated for 16 days. GM1 micelles contain 8 μM NBD-PE (green channel). All scale bars=10 μm

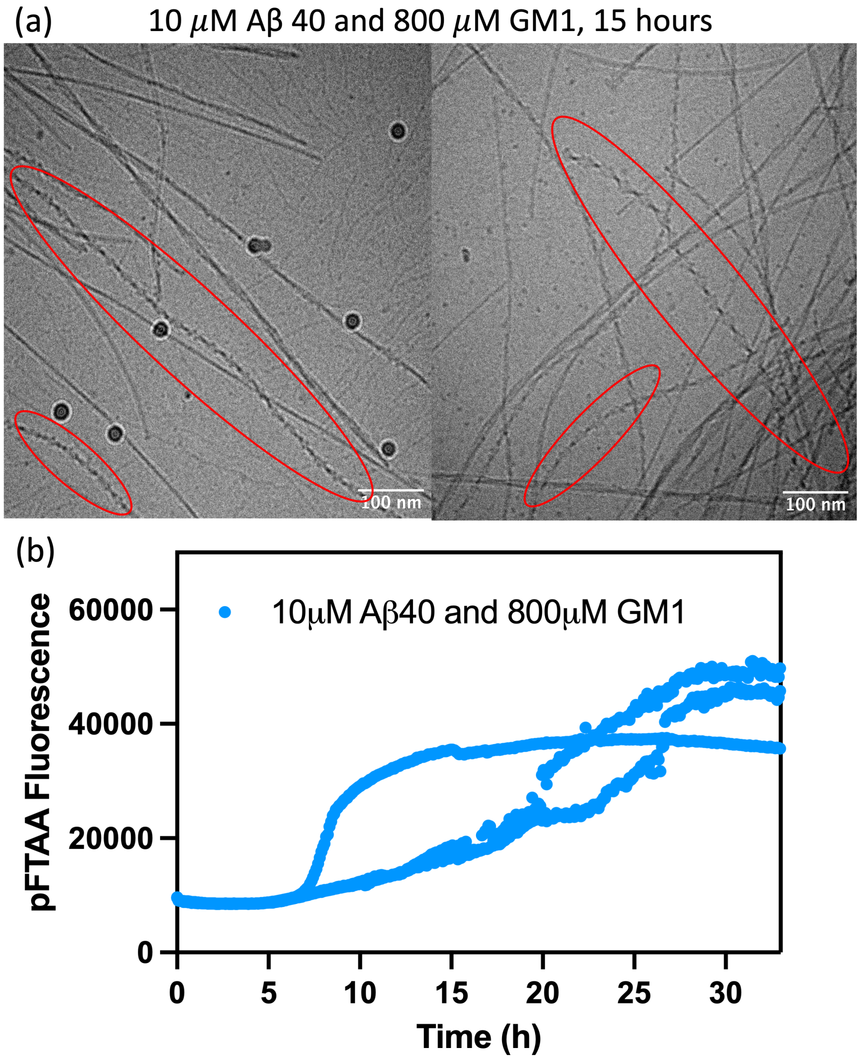

Figure S3: cryo-TEM images of the sample containing 10 μM Aβ40 and 800 μM GM1 after incubation for 15 hours. The red ovals enclose cases where lipids seem to be decorating Aβ fibrils (a). Aggregation kinetic of 10 μM Aβ40 and 800 μM GM1. The three curves shown are replicates of the same solution (b). All scale bars=100 nm.

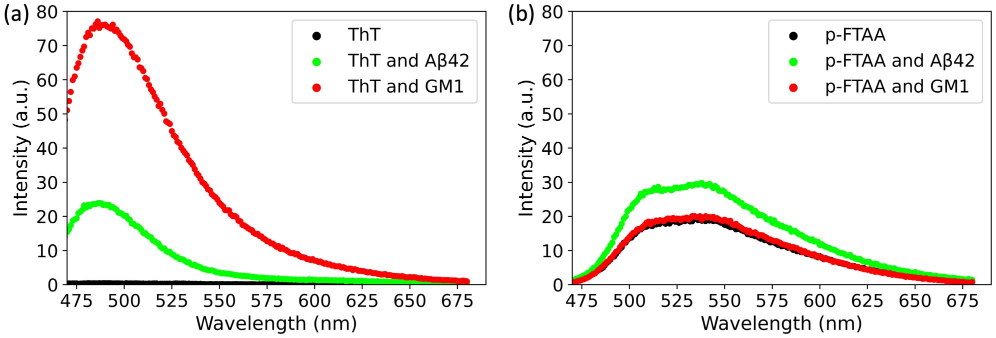

Figure S4: Fluorescence spectra of 20 μM ThT, a mixture of 20 μM ThT and 6 μM Aβ42 fibrils, and a mixture of 20 μM ThT and 400 μM GM1 (a). Fluorescence spectra of 1.5 μM pFTAA, mixture of 1.5 μM pFTAA and 14 μM Aβ42 fibrils, and mixture of 1.5 μM pFTAA and 360 μM GM1 (b).

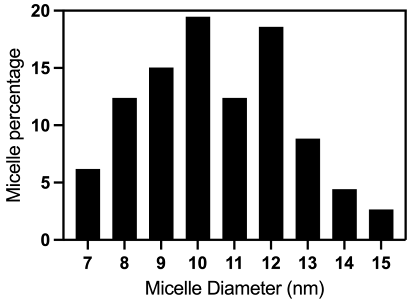

Figure S5: Analysis of GM1 micelle size based on the cryo-TEM images of 800 μM GM1 solutions. The percentage of counted GM1 micelles in cryo-TEM images is plotted against the micelle diameter. Measurements are performed for more than 100 objects

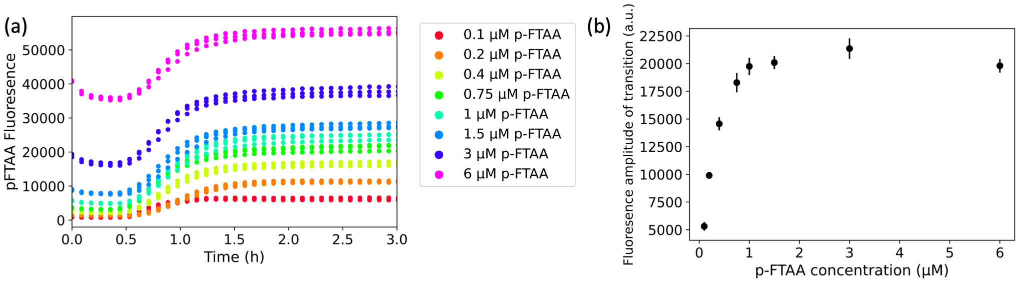

Figure S6: Aggregation kinetics of 5 μM Aβ42 with different concentration of pFTAA (a). The pFTAA intensity difference between 3 h and 0.4 h plotted against the pFTAA concentration (b).
